# Panapophenanthrin, a Rare Oligocyclic Diterpene from *Panus strigellus*

**DOI:** 10.3390/metabo13070848

**Published:** 2023-07-13

**Authors:** Natalia A. Llanos-López, Sherif Saeed Ebada, Aída M. Vasco-Palacios, Laura M. Sánchez-Giraldo, Lina López, Luisa F. Rojas, Attila Mándi, Tibor Kurtán, Yasmina Marin-Felix

**Affiliations:** 1 Department of Microbial Drugs, Helmholtz Centre for Infection Research (HZI) and German Centre for Infection Research (DZIF), DZIF Partner Site Hannover-Braunschweig, Inhoffenstrasse 7, 38124 Braunschweig, Germany; natalia.llanos@helmholtz-hzi.de; 2 Institute of Microbiology, Technische Universität Braunschweig, Spielmannstraße 7, 38106 Braunschweig, Germany; 3 Department of Pharmacognosy, Faculty of Pharmacy, Ain Shams University, Cairo 11566, Egypt; 4 Grupo de Microbiología Ambientaland Grupo BioMicro, Escuela de Microbiología, Universidad de Antioquia, Calle 70 No. 52-21, 050010 Medellin, Colombia; aida.vasco@udea.edu.co; 5 Grupo de Investigación de Biotecnología Industrial, Facultad de Ciencias, Universidad Nacional de Colombia Sede Medellín, Calle 59A No. 63-20, 050034 Medellin, Colombia; laumsanchezgir@unal.edu.co; 6 Grupo de Biotransformación, Escuela de Microbiología, Universidad de Antioquia, Calle 70 No. 52-21, 050010 Medellin, Colombia; linam.lopez@udea.edu.co (L.L.); lfernanda.rojas@udea.edu.co (L.F.R.); 7 Department of Organic Chemistry, University of Debrecen, P.O. Box 400, 4002 Debrecen, Hungary; mandi.attila@science.unideb.hu (A.M.); kurtan.tibor@science.unideb.hu (T.K.)

**Keywords:** basidiomycota, antimicrobial, cytotoxicity, secondary metabolites, white rot fungi

## Abstract

During the course of our search for biologically active secondary metabolites from fungal cultures, a new oligocyclic diterpenoidal derivative, panapophenanthrin (**1**), was isolated from *Panus strigellus*. In addition, two known metabolites, panepophenanthrin (**2**) and dihydrohypnophilin (**3**), were also obtained. The chemical structures of the isolated compounds were elucidated based on extensive 1D and 2D NMR spectral analyses together with high-resolution electrospray ionization mass spectrometry (HR-ESI-MS). The absolute configuration was determined through TDDFT-ECD calculations. All of the compounds were assessed for their antimicrobial and cytotoxic activities. Compounds **1** and **3** showed moderate to weak activities in the performed antimicrobial assays, while compound **1** exhibited potent cytotoxic activity against the mammalian cell lines mouse fibroblast (L929) and human endocervical adenocarcinoma (KB3.1).

## 1. Introduction

White rot fungi (WRF) are a broad class of wood-decaying basidiomycetes that have the ability to degrade lignin, a complex and recalcitrant component of plant cell walls [1]. These have been extensively studied for their capacity to break down and remediate organic contaminants, such as pharmaceutically active compounds (PhACs)—e.g., metoprolol and its recalcitrant metabolite metoprolol acid—due to their wide substrate spectrum and their capability to synthesize enzymatic complexes [2,3,4]. Other pollutants—including polychlorinated biphenyls (PCBs), polycyclic aromatic hydrocarbons (PAHs), petroleum hydrocarbons, and pesticides—have also been shown to be degraded by WRF [5,6,7].

The genus *Panus* belongs to the family Polyporaceae [8], and many of its studied species have been identified as WRF. This genus is defined by its dimitic hyphal system, with unbranched skeletal hyphae and lacking any binding processes, which makes it thin but tough [8]. It is also characterized by its agaricoid habit in the Polyporales [9]. Based on their morphological characteristics, Pegler combined both genera *Lentinus* and *Panus* into one large genus, *Lentinus* [10]. However, recent studies, including morphological, biological, and phylogenetic species concepts, have provided evidence to segregate it into two genera [11,12,13]. Studies also show that this group of lentinoid fungi needs further studies to elucidate the phylogenetic relationships between its sections. *Panus* includes species with skeletal hyphae (thick-walled, typically unbranched), lacking hyphal pegs, with metuloids and gloecystidia, and hymenophoral trama, mostly of a radiate construction [14]. Species of *Panus* are mainly widespread in tropical and subtropical regions, and typically grow on dead wood, downed logs, and tree stumps, playing a vital role in the decomposition of the organic material [8,9,15]. In Colombia, six species of *Panus* have been reported, including *P. conchatus*, *P. neostrigosus*, *P. rudis*, *P. similis*, *P. tephroleucus*, and *P. strigellus* [16]. Moreover, some of the species of *Panus* are edible and eaten in various cultures, including indigenous communities in the Amazon [17,18].

Although WRF have been extensively studied for their bioremediation capacities, little attention has been given to explore their potential for producing secondary metabolites. Different species of *Panus* have been found to produce diverse secondary metabolites, such as epoxy compound derivatives of quinones [19,20], sesquiterpenes [21], and other bioactive substances [22]; however, there are relatively few studies on their pharmacological properties and potential health benefits. Panepoxydone, a compound previously reported in *Lentinus crinitus*, was also found in *P. conchatus* and *P. rudis* and can interfere with the NF-κB mediated signal, which promotes tumor growth by inhibiting the phosphorylation of IκBα [19,23]. Another example is hexacyclinol, isolated from the fungal strain *P. rudis* HKI 0254, which exhibited antiproliferative activity on L-929 cells [24].

The genus *Panus* is a promising source of biologically active secondary metabolites due to its diverse chemical profile and potential therapeutic applications. Furthermore, the limited research conducted on this genus makes it a valuable source for the discovery of novel compounds. To explore this potential, we studied *P. strigellus* for the production of bioactive compounds. Our study led to the identification of a new polyhydro-4-oxa-monoepoxyphenanthrylen-1,7-dione derivative (**1**), along with the two known compounds, panepophenanthrin (**2**) [25], and a hirsutane sesquiterpenoidal congener, dihydrohypnophilin (**3**) [26,27]. The current paper provides a detailed report of the structural elucidation, antimicrobial activity, and cytotoxicity of the isolated compounds.

## 2. Materials and Methods

### 2.1. General Experimental Procedures

Nuclear magnetic resonance (NMR) spectra were recorded using an Avance III 500 MHz spectrometer equipped with a BBFO (plus) SmartProbe (^1^H 500 MHz, ^13^C 125 MHz; Bruker, Billerica, MA, USA) and an Avance III 700 MHz spectrometer equipped with a 5 mm TCI cryoprobe (^1^H 700 MHz, ^13^C 175 MHz; Bruker, Billerica, MA, USA) (sample temperature: 298 K). The NMR data were referenced to selected chemical shifts δ of CDCl_3_ (^1^H, δ = 7.27 ppm; ^13^C, δ = 77.2 ppm) and DMSO-*d6* (^1^H, δ = 2.50 ppm; ^13^C, δ = 39.51 ppm). 

Electrospray ionization mass (ESI-MS) spectra were recorded with an UltiMate^®^ 3000 Series uHPLC (Thermo Fisher Scientific; Waltman, MA, USA) employing a C18Acquity^®^ UPLC BEH column (2.1 × 50 mm, 1.7 μm; Waters, Milford, MA, USA) (temperature of the column: 40 °C), connected to an amaZon^®^ speed ESI-Iontrap-MS (Bruker; Billerica, MA, USA). The following parameters were used to set up the HPLC system: solvent A: Deionized H_2_O + 0.1% formic acid (FA) (*v*/*v*), solvent B: acetonitrile (MeCN) + 0.1% FA (*v*/*v*) as the mobile phase; gradient: 5% B for 0.5 min, increasing to 100% B in 19.5 min and maintaining isocratic conditions at 100% B for 5 min; flow rate: 0.6 mL/min, and Diode-Array Detection (DAD) at 190–600 nm. The crude extracts and pure compounds were dissolved in a solution of acetone and methanol (1:1) to achieve a concentration of 4.5 mg/mL and 1 mg/mL, respectively. High-resolution electrospray ionization mass spectrometry (HR-ESI-MS) spectra were obtained with an Agilent 1200 Infinity Series HPLC–UV system (Agilent Technologies, Böblingen, Germany) with the same conditions as for ESI-MS spectra, connected to a maXis^®^ ESI-TOF mass spectrometer (Bruker; Daltonics, Bremen, Germany)) (scan range 100–2500 *m/z*, capillary voltage 4500 V, dry temperature 200 °C).

Optical rotations (OR) were recorded in chloroform or DMSO (Uvasol, Merck; Darmstadt, Germany) using a MCP-150 polarimeter (Anton Paar; Seelze, Germany) at 20 °C. UV/Vis spectra measurements were carried out using the UV-Vis spectrophotometer UV-2450 (Shimadzu; Kyoto, Japan), while electronic circular dichroism (ECD) spectra were collected with a J-815 spectropolarimeter (Jasco, Pfungstadt, Germany).

### 2.2. Fungal Isolation

The specimen was collected in the municipality of Santa Fe de Antioquia, located in the department of Antioquia, Colombia (6.56° N 75.83° W, 571 masl), with an average temperature of 28 °C. Sporomes of *Panus* were collected using an opportunistic approach of convenience sampling [28]. Macromorphological characters were described, including the fresh color, according to the Methuen Handbook of Colour [29]. Specimens were dried in a food dehydrator and placed in plastic bags for transport. All collections were deposited in the University of Antioquia herbarium (HUA). Isolation and culture were performed using small fragments of the pileus. The obtained pure cultures were preserved in potato dextrose agar medium (PDA agar, Himedia, Mumbai, India) at 4 °C until the beginning of the experiments. The strain was deposited in the Microorganism Collection of the School of Microbiology CM-EM-UdeA (CM-UDEA-H9, voucher basidiomata 2574a AMV).

### 2.3. DNA Extraction, PCR Amplification and Sequencing

DNA of *P. strigellus* was extracted from a 1-week-old colony growing on yeast malt agar (YM agar, malt extract 10 g/L, yeast extract 4 g/L, D-glucose 4 g/L, agar 20 g/L, pH 6.3 before autoclaving) according to the Fungal gDNA Miniprep Kit EZ-10 Spin Column kit protocol (NBS Bio-logicals, Cambridgeshire, UK). The polymerase chain reaction (PCR) was performed to amplify partial sequences of DNA regions, i.e., the internal transcribed spacer region (ITS) using the standard primers ITS1F [30] and ITS4 [31], and the 28S large subunit (LSU) with the primers LR0R and LR7 [32]. The PCR products were purified and sequenced using the Sanger Cycle Sequencing method at Microsynth Seqlab GmbH (Göttingen, Germany). Consensus sequences were obtained using the Geneious^®^ 7.1.9 program [33]. Afterward, the sequences were compared to the available reference data using the Basic Local Alignment Search Tool (BLAST, https://blast.ncbi.nlm.nih.gov/Blast.cgi accessed on 7 June 2023) to achieve the identification of the fungus as *P. strigellus*. The ITS sequence shows affinities to *P. strigellus* (99.76% nucleotide similarity with JQ955727, and 99.05% with MT669136 and JQ955724), while the LSU sequence showed 99.25% nucleotide similarity with *P. conchatus* (ON417226) and 99.17% with *P. lecomtei* (KP135233). However, the morphological study of the voucher specimen, 2574a AMV, from which the strain was isolated, is conclusive with the tropical species *P. strigellus*. The sequences generated in this study were deposited in GenBank (ITS: OR160301, LSU: OR165097).

### 2.4. Fermentation and Extraction

The fungal strain was subjected to submerged culture in shaker flasks. A 5-L fermentation was carried out in twenty-five shaker flasks of 500 mL Erlenmeyer shape culture flasks containing 200 mL of YM medium (10 g/L malt extract, 4 g/L d-glucose, 4 g/L yeast extract, pH 6.3 before autoclaving). For the inoculum, a well-grown mycelium on an YM agar plate was cut into small pieces using a cork borer (1 cm × 1 cm) and six plugs were inoculated in each flask. The cultures were incubated under shake conditions in the dark at 140 rpm and 23 °C. The fermentation was monitored by checking the concentration of free glucose with Medi-Test glucose (Macherey-Nagel, Düren, Germany). The free glucose was fully consumed after 11 days, and the fermentation was terminated after 3 days of glucose depletion.

To extract the secondary metabolites from the culture, the supernatant and mycelium were first separated through vacuum filtration. The supernatant was decanted with an equal amount of ethyl acetate in a separatory funnel. The organic phase obtained was filtered through anhydrous sodium sulfate and the permeate was evaporated to dryness in vacuo at 40 °C with a rotary evaporator (Heidolph Instruments GmbH and Co. KG, Schwabach, Germany; pump: Vacuubrand GmbH and Co. KG, Wertheim am Main, Germany) to obtain the crude extract. To extract the secondary metabolites from the mycelium, it was initially soaked with acetone and sonicated for 30 min at 40 °C in an ultrasonic bath (Sonorex Digital 10 P, Bandelin Electronic GmbH and Co. KG, Berlin, Germany); the acetone was evaporated in vacuo at 40 °C, and the resulting aqueous phase was decanted with an equal amount of ethyl acetate. To obtain the crude extract, the organic phase was filtered through anhydrous sodium sulfate and then evaporated to dryness. The process was conducted twice, yielding 2565 mg of supernatant extract and 1824 mg of mycelium extract. 

### 2.5. Isolation of Compounds **1**–**3**

For a further separation of the compounds, 904 mg of the supernatant extract were dissolved in methanol, portioned in 4 × 226 mg, and fractionated using a preparative reverse phase HPLC (Büchi, Pure C-850, 2020, Flawil, Switzerland). A Gemini^®^ 10 μm C18 110 Å column (250 × 50 mm; Phenomenex, Torrance, CA, USA) was used as the stationary phase. Deionized H_2_O + 0.1% formic FA (*v*/*v*) (solvent A) and acetonitrile (MeCN) + 0.1% FA (*v*/*v*) (solvent B) were used as the mobile phase with a flow rate of 40 mL/min. The separation was carried out with an elution gradient started with isocratic conditions at 5% solvent B for 8 min, followed by a gradual increase to 20% B in 5 min, then an increase from 20% B to 30% B in 30 min, 30% B to 42%B in 30 min, 42% B to 100% B in 5 min, and finally isocratic conditions at 100% B for 5 min. UV detection was performed at 210, 254, 300, and 350 nm and eight fractions (F1–F8) were collected based on the observed peaks. The purity of the fractions was checked using HPLC-DAD-ESI-MS.

Fraction F5 (74.8 mg) was further separated using the same equipment and mobile phase as before, but with a flow rate of 20 mL/min and using a Gemini^®^ 10 μm C18 110 Å column (250 × 21.2 mm; Phenomenex, Torrance, CA, USA) as the stationary phase. For F5, the gradient was operated with isocratic conditions at 10% B for 5 min, followed by an increase from 10% B to 20% B in 10 min, from 20% B to 25% B in 20 min, 25% B to 40% B in 15 min, 40% B to 100% B in 5 min, and a final isocratic step of 100% B for 5 min. Ten sub-fractions (G1–G10) were collected from this separation. Compound **1** (0.59 mg, *t*_R_ = 32–33 min) was obtained from G5 (19.8 mg) through preparative reverse phase HPLC (Büchi, Pure C-850, 2020, Flawil, Switzerland) with a Synergi^TM^ 10 µm Polar-RP 80 Å column (250 × 50 mm; Phenomenex, Torrance, CA, USA). The aforementioned solvents A and B were employed as the mobile phase with a flow rate of 20 mL/min and a gradient of 5% B for 3 min, then an increase from 5% B to 15% B in 18 min, 15% B to 100% B in 25 min, and finally isocratic conditions at 100% B for 5 min. Sub-fraction G6 (17.7 mg) was further separated using the same instruments and conditions as G5, beginning with isocratic conditions at 5% B for 3 min, afterward an increase to 50% B in 25 min, then an increase to 100% B in 8 min, finished with isocratic conditions at 100% B for 3 min. Four sub-fractions (H1–H4) were obtained from this separation. A total of 10.34 mg of H4 were further purified through preparative reverse phase HPLC (Büchi, Pure C-850, 2020, Flawil, Switzerland) using a Luna^®^ 5 μm C18 110 Å column (250 × 21.2 mm; Phenomenex, Torrance, CA, USA) as the stationary phase, and the same solvents, A and B, as the mobile phase with a flow rate of 15 mL/min. The gradient was operated with isocratic conditions at 10% B for 3 min, then an increase from 10% B to 25% B in 36 min, from 25% B to 100% B in 3 min, and a final isocratic step of 100% B for 3 min. From this separation, compound **2** (3.49 mg, *t*_R_ = 27.5–28.5 min) was isolated.

Fraction F6 (50.2 mg) was fractionated using a preparative reverse phase HPLC (Büchi, Pure C-850, 2020, Flawil, Switzerland). A Gemini^®^ 10 μm C18 110 Å column (250 × 21.2 mm; Phenomenex, Torrance, CA, USA) was employed as the stationary phase. Solvents A and B were used as the mobile phase with a flow rate of 20 mL/min. For the separation, the elution gradient started with isocratic conditions at 25% B for 5 min, followed by an increase to 45% B in 20 min, then an increase to 100% B in 10 min, and ended with isocratic conditions at 100% B for 5 min. Three sub-fractions (J1–J3) were collected and J3 (21 mg) was further separated to obtain compound **3** (4.51 mg, *t*_R_ = 19.5–20.5 min) using the same equipment and XBridge 5 μm C18 column (250 × 19 mm; Waters, Milford, MA, USA) as the stationary phase. Solvents A and B were employed as the mobile phase with a flow rate of 20 mL/min. In order to separate sub-fraction J3, the elution gradient was initiated by maintaining the isocratic conditions at 5% B for 3 min, followed by an increase to 20% B in 2 min, 20% B to 30% B in 30 min, 30% B to 100% B in 5 min, and ending with isocratic conditions at 100% B for 3 min. Further details about the purification data are available in Supplementary Materials (Figure S12).

#### 2.5.1. Panapophenanthrin (**1**)

White solid powder; 0.59 mg; ${\left[\alpha \right]}_{D}^{20}$ –7 (c 0.04, chloroform); UV/Vis (MeOH): λ_max_ (log ε) = 246 (0.1), 202.0 (0.3) nm; ECD (MeOH, λ (nm) (Δε), c 4.97 × 10^−4^ M): 322 (−1.88), 306 (+3.06), 206 (−7.98); NMR data (^1^H NMR: 500 MHz, ^13^C NMR: 125 MHz) see Table 1; HR-(+)ESIMS: m/z 403.1750 [M+H]^+^ (calcd. 403.1751 for C_22_H_27_O_7_^+^), 425.1568 [M+Na]^+^ (calcd. 425.1571 for C_22_H_26_NaO_7_^+^), 827.3248 [2M+Na]^+^ (calcd. 827.3249 for C_44_H_52_NaO_14_^+^); t_R_ = 6.72 min (HR-LC-ESIMS). C_22_H_26_O_7_ (402.11 g/mol).

#### 2.5.2. Panepophenanthrin (**2**)

White solid powder; 3.49 mg; ${\left[\alpha \right]}_{D}^{20}$ +57 (*c* 1.0, DMSO); UV/Vis (MeOH): λ_max_ (log *ε*) = 256 (0.1), 203.0 (0.2) nm; NMR data (^1^H NMR: 500 MHz, ^13^C NMR: 125 MHz in DMSO-*d_6_*) see Supplementary Material Table S1; HR-(+)ESIMS: *m/z* 443.1674 [M+Na]^+^ (calcd. 443.1676 for C_22_H_28_NaO_8_^+^), 863.3460 [2M+Na]^+^ (calcd. 863.3461 for C_44_H_56_NaO_16_^+^); *t*_R_ = 3.90 min (HR-LC-ESIMS). C_22_H_28_O_8_ (420.12 g/mol).

#### 2.5.3. Dihydrohypnophilin (**3**)

White solid powder; 4.51 mg; ${\left[\alpha \right]}_{D}^{20}$ +155 (*c* 0.1, chloroform); UV/Vis (MeOH): λ_max_ (log *ε*) = 202.0 (0.3) nm; NMR data (^1^H NMR: 500 MHz, ^13^C NMR: 125 MHz in chloroform-*d*) see Supplementary Material Table S2; HR-(+)ESIMS: *m/z* 233.1532 [M-H_2_O+H]^+^ (calcd. 233.1536 for C_15_H_21_O_2_^+^), *m/z* 251.1637 [M+H]^+^ (calcd. 273.1461 for C_15_H_22_NaO_3_^+^), 501.3208 [2M+H]^+^ (calcd. 501.3211 for C_30_H_45_O_6_^+^), 523.3027 [2M+Na]^+^ (calcd. 523.3030 for C_30_H_22_NaO_6_^+^); *t*_R_ = 5.20 min (HR-LC-ESIMS). C_15_H_22_O_3_ (250.12 g/mol).

### 2.6. Antimicrobial Assay

The Minimum Inhibitory Concentration (MIC) of the isolated compounds was determined following the method described by Charria-Girón et al. [34]. Compounds **2** and **3** were tested against the fungi *Schizosaccharomyces pombe, Pichia anomala, Mucor hiemalis Candida albicans*, and *Rhodotorula glutinis*; the Gram-positive bacteria *Bacillus subtilis, Mycobacterium smegmatis*, and *Staphylococcus aureus*; and the Gram-negative bacteria *Acine-tobacter baumanii, Chromobacterium violaceum, Escherichia coli*, and *Pseudomonas aeruginosa.* Compound **1** was tested against *M. hiemalis, B. subtilis, E. coli, Ps. aeruginosa*, and *S. aureus.* A serial dilution assay was performed in 96-well microtiter plates, using MYC medium (1% bactopeptone, 1% yeast extract, 2% glycerol, pH 6.3) for fungi and adjusted at OD_548_ nm to 0.1. MHB medium (Müller-Hinton Broth: SNX927.1, Carl Roth GmbH, Karlsruhe, Germany) was used for bacteria and most of the cell suspensions were adjusted at OD_600_ nm to 0.1. Ciprofloxacin, oxytetracycline, kanamycin, and gentamycin were used as positive controls against bacterial pathogens, while nystatin was used as the positive control against fungi. The MIC was determined as the lowest concentration of a compound needed to prevent the visible growth of the test organism under specific conditions. 

### 2.7. Cytotoxicity Assay

The in vitro cytotoxicity of the isolated metabolites was evaluated against the two mammalian cell lines, KB 3.1 (human endocervical adenocarcinoma) and L929 (mouse fibroblasts), in a 96-well microtiter plate. The IC_50_ was determined using the colorimetric tetrazolium dye MTT (3-(4,5-dimethylthiazol-2-yl)-2,5-diphenyltetrazolium bromide) assay with epothilone B as a positive control, following the experimental procedure described by Charria-Girón et al. [34]. The half-maximum inhibitory concentration (IC_50_)—the concentration at which cell growth inhibition reached 50% compared to the control—was calculated.

### 2.8. Computational Section

Mixed torsional/low-mode conformational searches were carried out by means of the Macromodel 10.8.011 software, using the MMFF with an implicit solvent model for CHCl_3_ and applying a 21 kJ/mol energy window [35]. Geometry re-optimizations of the resultant conformers (ωB97X/TZVP PCM/MeOH) and TDDFT-ECD (B3LYP/TZVP PCM/MeOH, BH&HLYP/TZVP PCM/MeOH, CAM-B3LYP/TZVP PCM/MeOH and PBE0/TZVP PCM/MeOH) calculations were performed with the Gaussian 09 package [36]. ECD spectra were generated as sums of Gaussians with 4200 cm^−1^ width at half-height, using dipole-velocity-computed rotational strength values [37]. Boltzmann distributions were estimated from the DFT energies. Visualization of the results was performed using the MOLEKEL 5.4 software package [38]. 

## 3. Results and Discussion

### 3.1. Isolation and Identification of Compounds (**1**–**3**)

**Figure 1:**
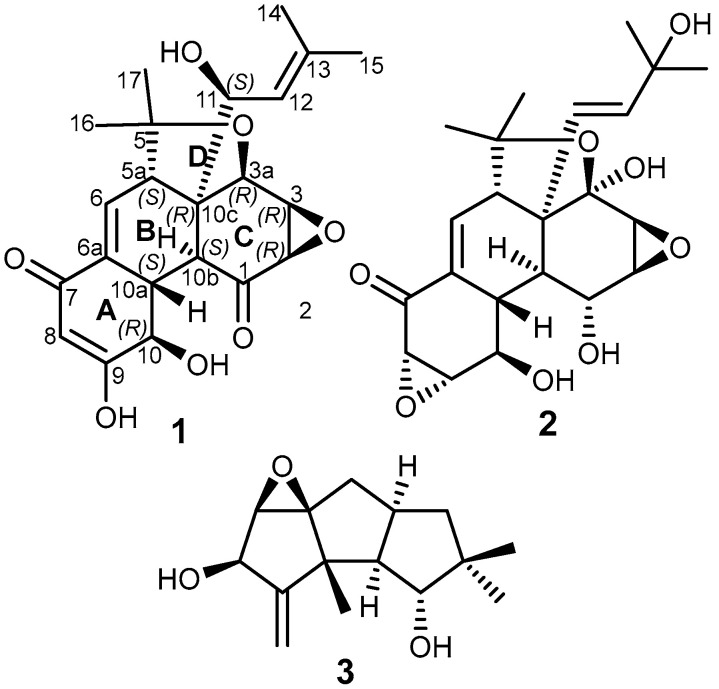
Chemical structures of **1**–**3**.

**Figure 2:**
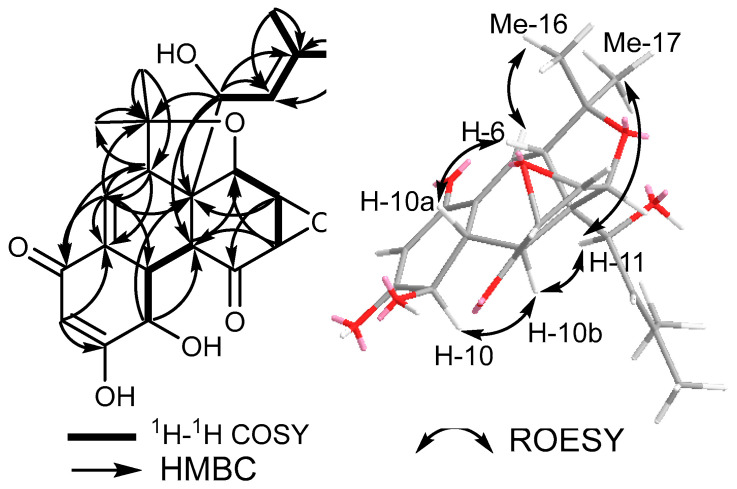
Key COSY, HMBC and ROESY correlations of **1**.

Compound **1** was purified as a white solid powder that revealed a quasi-molecular ion peak at *m/z* 403.1750 [M+H]^+^ (calculated 403.1751) and at *m/z* 425.1568 [M+Na]^+^ (calculated 425.1571), establishing its molecular formula as C_22_H_26_O_7_ and indicating its inclusion of ten degrees of unsaturation. The ^13^C NMR and HSQC spectral data of **1** (Table 1) unveiled the presence of twenty-two different carbon resonances that can be distinguished into seven quaternary carbon atoms, including two ketocarbonyl groups at δ_C_ 201.2 (C-1) and δ_C_ 193.9 (C-7) and three olefinic carbons at δ_C_ 165.2 (C-9), δ_C_ 142.8 (C-13), and at δ_C_ 133.3 (C-6a), along with two aliphatic quaternary carbons at δ_C_ 81.3 (C-5) and δ_C_ 59.6 (C-10c). In addition, the ^1^H, ^13^C NMR, and HSQC spectral data of **1** (Table 1) revealed the presence of three olefinic protons at δ_H_ 5.03 (dt, *J =* 10.1, 1.5 Hz, H-12; δ_C_ 120.0), δ_H_ 6.52 (dd, *J =* 4.1, 2.8 Hz, H-6; δ_C_ 134.4), and a downfield proton at δ_H_ 8.08 (d, *J =* 2.0 Hz, H-8; δ_C_ 129.5), together with eight aliphatic methines, four methyls recognized into two allylic methyls at δ_H_ 1.78 and δ_H_ 1.76 that were directly correlated to two carbons at δ_C_ 18.5 (C-14) and δ_C_ 26.4 (C-15), respectively, and two singlet methyl groups at δ_H_ 1.09 (H_3_-17; δ_C_ 23.7) and δ_H_ 1.48 (H_3_-16; δ_C_ 28.3). By comparing the obtained results with the reported literature and the NMR data obtained for **2** (see Supplementary Material Table S1), compound **1** was suggested to be a related derivative to panepophenanthrin (**2**) and hexacyclinol, previously reported from different strains of the fungus *P. rudis* [16,17]. A careful investigation of the 2D NMR spectral data of **1** and **2** gave sufficient proofs interpreting the differences in their depicted chemical structures (Figure 1). The ^1^H-^1^H COSY and HSQC spectra of **1** (Figure 2, see Supplementary Materials Figures S4 and S6) revealed four spin systems: the first comprises one olefinic proton (H-6) and one aliphatic methine proton at δ_H_ 3.52 (t, *J =* 3.7 Hz, H-5a; δ_C_ 50.3); the second extends over three methine protons at δ_H_ 4.29 (t, *J =* 5.2 Hz, H-10; δ_C_ 68.6), δ_H_ 2.65 (ddd, *J =* 11.0, 5.7, 2.8 Hz, H-10a; δ_C_ 44.8), and at δ_H_ 2.38 (dd, *J =* 11.0, 1.6 Hz, H-10b; δ_C_ 47.5); the third was defined among the other three aliphtic methines at δ_H_ 5.13 (br s, H-2; δ_C_ 64.0), δ_H_ 3.58 (m, *J =* 1.6 Hz, H-3; δ_C_ 55.7), and at δ_H_ 3.66 (dd, *J =* 3.6, 1.3 Hz, H-3a; δ_C_ 60.4); and the fourth spin system was distinguished between one oxygenated aliphatic methine at δ_H_ 5.10 (d, *J =* 10.0 Hz, H-11; δ_C_ 80.3), H-12, and also exhibiting long range correlations to two allyl methyl groups (Me-14 and Me-15). The HMBC spectrum of **1** (Figure 2) revealed key correlations from H-6 and H-8 to C-6a, whereas H-6 revealed a HMBC correlation to the ketocarbonyl carbon at C-7, and H-8 revealed a key correlation to C-9, indicating the inclusion of ring A to *α*,*β*-unsaturated carbonyl moiety. Further key HMBC correlations were identified from H-3, along with long range correlations from H-11 to a ketocarbonyl carbon at C-1 and in addition to the key correlations from the two singlet methyl groups (Me-16 and Me-17) to C-5 (δ_C_ 81.3) and C-5a (δ_C_ 50.3). Based on the aforementioned results, compound **1** was identified as a new polyhydro-4-oxa-monoepoxyphenanthrylen-1,7-dione related to panepophenanthrin (**2**) and hexacyclinol that was trivially named as panapophenanthrin.

The relative stereochemistry of **1** was determined based on its ROESY spectrum (Figure 2), which revealed key NOE correlations between H-10, H-10b, H-11, and Me-17, indicating that they are facing the same plane of the molecule; on the other hand, other NOE correlations were noticed among H-6, H-10a, and Me-16, supporting their presence toward an opposite plane of the molecule.

**Figure 3:**
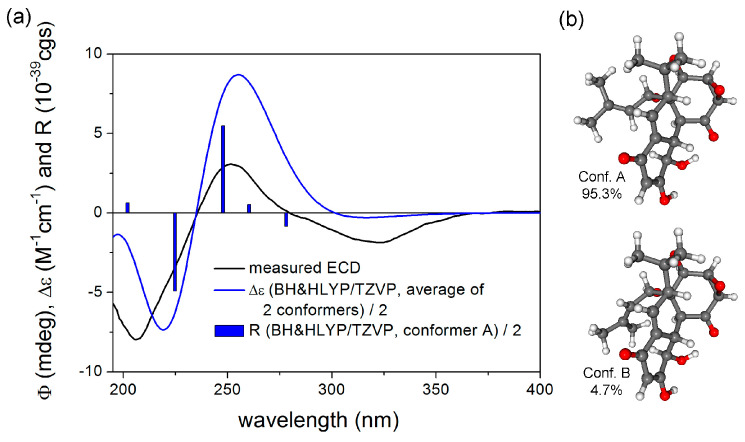
(**a**) Experimental ECD spectrum of **1** in MeOH compared with the BH&HLYP/TZVP PCM/MeOH ECD spectrum of (2*R*,3*R*,3a*R*,5a*S*,10*R*,10a*S*,10b*S*,10c*R*,11*S*)-**1** calculated for the ωB97X/TZVP PCM/MeOH conformers. (**b**) Low-energy ωB97X/TZVP PCM/MeOH conformers of (2*R*,3*R*,3a*R*,5a*S*,10*R*,10a*S*,10b*S*,10c*R*,11*S*)-**1**.

In order to elucidate the absolute configuration, the TDDFT-ECD approach was applied on the (2*R*,3*R*,3a*R*,5a*S*,10*R*,10a*S*,10b*S*,10c*R*,11*S*) enantiomer of **1** [39,40]. The initial Merck Molecular Force Field (MMFF) conformational search resulted in two conformer clusters with a 21 kJ/mol energy window, the ωB97X/TZVP [41] PCM/MeOH re-optimization of which yielded conformers A and B with Boltzmann populations of 95.3% and 4.7%, respectively. These conformers differed only in the orientation of the C-10c substituent. The Boltzmann-averaged ECD spectra calculated (Figure 3) at various levels reproduced all the transitions of the experimental spectrum, allowing the determination of the absolute configuration as (2*R*,3*R*,3a*R*,5a*S*,10*R*,10a*S*,10b*S*,10c*R*,11*S*).

### 3.2. Biological Assays

To assess the antimicrobial activity of compounds **1**–**3**, a serial dilution assay was conducted against several Gram-positive and Gram-negative bacteria, as well as fungal strains, namely: *Schizosaccharomyces pombe, Pichia anomala, Mucor hiemalis Candida albicans, Rhodotorula glutinis, Bacillus subtilis, Mycobacterium smegmatis, Staphylococcus aureus, Acinetobacter baumanii, Chromobacterium violaceum, Escherichia coli,* and *Pseudomonas aeruginosa.* The results obtained (Table 2) revealed that panapophenanthrin (**1**) and dihydrohypnophilin (**3**), rather than panepophenanthrin (**2**), have only moderate to weak antimicrobial activities, with MICs ranging between 33.3 and 66.6 µg/mL. Compound **1** exhibited moderate activity against the Gram-positive bacterium *B. subtilis,* with a MIC value of 33.3 µg/mL, compared to oxytetracyclin, which was used as the positive control. In addition, **1** inhibited the growth of the Gram-positive bacterium *S. aureus* at 66.7 µg/mL, indicating weak activity in comparison to the positive control, gentamycin. Compound **2** was inactive against all of the organisms tested. It is important to note that **1** exhibited antimicrobial activity despite being a derivative of **2**, while **2** demonstrated inactivity. This intriguing result highlights a difference in the biological properties between the two compounds. However, it is difficult to identify the functional group responsible for the observed activity in **1** based on the structural differences with compound **2**. Therefore, further studies are needed to determine the group contributing to the antimicrobial activity of **1**. On the other hand, compound **3** showed weak activity against *B. subtilis*, with a MIC value of 66.7 μg/mL, compared to the positive control. However, it was inactive against the remaining organisms tested.

Compound **2** was originally isolated from the fermented broth of *P. rudis* FO 8994 [25] and it has been extensively studied for its capacity to inhibit the ubiquitin-activating enzyme (E1) [25,42,43]. It was the first reported inhibitor of E1 from a natural source. The enzyme E1 plays an indispensable role in the ubiquitin-proteasome pathway (UPP), which regulates diverse cellular processes through the degradation of targeted proteins [42,43]. The abnormal functioning of this pathway has been associated with neurodegenerative disease and human cancers [44,45]. Moreover, **2** has generated significant interest among synthetic chemists owing to its unique molecular architecture, distinguished by a densely substituted tetracyclic core. This core structure is notable for its 11 contiguous stereocenters, including two quaternaries [42]. The unique structural characteristics of this molecule, together with its significant biological activity, have prompted extensive studies on its total synthesis [42,43,46,47]. Compound **2**, along with its related derivative **1**, belong to a general class of related epoxyquinoid natural products that are synthesized through Diels–Alder-type dimerization [43]. This group of molecules exhibits a degree of structural complexity spanning from the lower order epoxyquinols—including (+)-isoepoxydon, (+)- epiepoformin, and (+)-bromoxone—to its acetylated derivative [47]. Compounds from this class have been previously isolated from phylogenetically diverse organisms, such as fungi, bacteria, and worms, inhabiting a wide range of terrestrial and marine ecosystems [47].

Compound **3**, a sesquiterpene with a hirsutane skeleton, was first isolated from the fungus *L. crinitus* [26]. It is structurally related to the hirsutic acid C and is characterized by the presence of an α-methylene ketone moiety [26]. In a prior study conducted by Abate and Abraham (1994), the compound was tested against different microorganisms, revealing activity against *Bacillus cereus* and spores of *Aspergillus flavus*, *Aspergillus niger*, and *Mucor rouxii* [26]. 

On the other hand, panapophenanthrin (**1**) exhibited potent cytotoxic activity against two mammalian cell lines—mouse fibroblast (L929) and human endocervical adenocarcinoma (KB3.1)—compared to its related derivative **2** (Table 3). This imparts a possible role for their structural differences in eliciting cytotoxic activity. Dihydrohypnophilin (**3**) revealed very weak to no cytotoxic activity against the tested cell lines.

Although compound **3** showed weak cytotoxic activity in our experiment, Abate and Abraham previously reported an IC_50_ of 2.4 g/mL of **3** against the L929 cell line [26]. Moreover, prior studies have reported the strong activity of **3** against the malarial parasite *Plasmodium falciparum,* and cytotoxic activity against the human small lung cancer (NCI-H187) cell line and the derived cells from the kidney of an African green monkey (Vero) [27].

To the best of our knowledge, compound **2** has solely been reported from *P. rudis*, highlighting its possible significance in the genus. Compound **3**, on the other hand, has only been isolated from other genera, but all belonging to the family Polyporaceae, such as *L. crinitus* [26], *L. conatus* [27], *L. strigosus* [48], and *Cerrena* sp. A593 [49]. However, the boundaries between *Lentinus* and *Panus* remain unclear, and species of both genera are still awaiting proper classification based on polyphasic studies [13,14]. Therefore, it is possible that the compounds reported have been associated with the incorrect genus due to this problem. Further research is needed to explore the chemical diversity and biological activities of secondary metabolites from both genera, as well as to understand their evolutionary differentiation.

## 4. Conclusions

In the current study, three compounds—including one novel compound and two known compounds—were isolated from the submerged culture of *P. strigellus.* The novel compound was named panapophenanthrin (**1**), while the known compounds were identified as panepophenanthrin (**2**) and dihydrohypnophilin (**3**). Our results provide new insights into the chemical diversity of the fungus *P. strigellus.* According to our research findings, compounds **1** and **2** belong to a rare category of oligocyclic terpenoidal metabolites that are only known from the genus *Panus*. The discovery of this novel compound highlights the potential of the genus *Panus* as a source of bioactive substances. In addition, this finding shows the importance of studying unexplored tropical species for the discovery of novel natural products. Further studies are needed to fully characterize the bioactivity of these compounds and to explore their potential applications.

## Figures and Tables

**Table 1:** ^1^H and ^13^C NMR data of **1**.

Pos.	δ_H_ (Multi, *J* [Hz]) ^a^	δ_C_, Type ^b,e^	δ_H_ (Multi, *J* [Hz]) ^c^	δ_C_, Type ^d,e^
1		201.2, CO		202.9, CO
2	5.13 (br s, 1H)	64.0, CH	5.17 (br s, 1H)	64.5, CH
3	3.58 (m, *J* = 1.6 Hz, 1H)	55.7, CH	3.50 (m, overlapped, 1H)	57.8, CH
3a	3.66 (dd, *J* = 3.6, 1.3 Hz, 1H)	60.4, CH	3.61 (dd, *J* = 3.8, 1.2 Hz, 1H)	62.5, CH
5		81.3, C		83.0, C
5a	3.52 (t, *J* = 3.7 Hz, 1H)	50.3, CH	3.50 (t, *J* = 3.2 Hz, 1H)	50.0, CH
6	6.52 (dd, *J* = 4.1, 2.8 Hz, 1H)	134.4, CH	6.31 (dd, *J* = 4.8, 2.8 Hz, 1H)	133.2, CH
6a		133.3, C		136.6, C
7		193.9, CO		198.0, CO
8	8.08 (d, *J* = 2.0 Hz, 1H)	129.5, CH	8.56 (s, 1H)	130.9, CH
9		165.2, C		170.3, C
10	4.29 (t, *J* = 5.7 Hz, 1H)	68.6, CH	4.12 (d, *J* = 5.7 Hz, 1H)	70.5, CH
10a	2.65 (ddd, *J* = 11.0, 5.7, 2.8 Hz, 1H)	44.8, CH	2.59 (ddt, *J* = 11.2, 5.7, 2.8 Hz, 1H)	45.9, CH
10b	2.38 (dd, *J* = 11.0, 1.6 Hz, 1H)	47.5, CH	2.46 (dt, *J* = 11.2, 1.6 Hz, 1H)	48.4, CH
10c		59.6, C		61.5, C
11	5.10 (d, *J* = 10.0 Hz, 1H)	80.3, CH	5.56 (d, *J* = 9.9 Hz, 1H)	80.8, CH
12	5.03 (dt, *J* = 10.0, 1.4 Hz, 1H)	120.0, CH	4.98 (dt, *J* = 9.9, 1.4 Hz, 1H)	123.1, CH
13		142.8, C		140.0, C
14	1.78 (d, *J* = 1.4 Hz, 3H)	18.5, CH_3_	1.76 (d, *J* = 1.4 Hz, 3H)	19.1, CH_3_
15	1.76 (d, *J* = 1.4 Hz, 3H)	26.4, CH_3_	1.71 (d, *J* = 1.4 Hz, 3H)	26.6, CH_3_
16	1.48 (s, 3H)	28.3, CH_3_	1.42 (s, 3H)	30.2, CH_3_
17	1.09 (s, 3H)	23.7, CH_3_	1.06 (s, 3H)	25.9, CH_3_

Measured in chloroform-*d* at ^a^ 500 (for ^1^H) and ^b^ 125 (for ^13^C) MHz. Measured in methanol-*d_4_*: acetone-*d_6_* (3:1) at ^c^ 500 (for ^1^H) and ^d^ 125 (for ^13^C) MHz. ^e^ Assigned based on HMBC and HSQC spectra.

**Table 2:** Minimum inhibitory concentration (MIC) of compounds **1**–**3** against test organisms.

MIC (µg/mL)				
Test Organism	1	2	3	Reference
*Schizosaccharomyces pombe*	n.t	-	-	4.2 ^N^
*Pichia anomala*	n.t	-	-	8.3 ^N^
*Mucor hiemalis*	-	-	-	4.2 ^N^
*Candida albicans*	n.t	-	-	4.2 ^N^
*Rhodotorula glutinis*	n.t	-	-	2.1 ^N^
*Acinetobacter baumanii*	n.t	-	-	0.52 ^C^
*Escherichia coli*	-	-	-	0.83 ^G^
*Bacillus subtilis*	33.3	-	66.6	16.6 ^O^
*Mycobacterium smegmatis*	n.t	-	-	1.7 ^K^
*Staphylococcus aureus*	66.6	-	-	0.21 ^G^
*Pseudomonas aeruginosa*	-	-	-	0.21 ^G^
*Chromobacterium violaceum*	n.t	-	-	0.83 ^G^

(-): no inhibition observed, n.t: not tested, ^C^: Ciprobay 2.54 mg/mL, ^G^: Gentamycin 1 mg/mL, ^K^: Kanamycin 1 mg/mL, ^N^: Nystatin 10 mg/mL, ^O^: Oxytetracyclin 1 mg/mL.

**Table 3:** Cytotoxic activity (IC_50_) of compounds **1**–**3** against mammalian cell lines.

IC_50_ (µM)				
Cell Lines	1	2	3	Epothilone B
L929	13.2	*	103.9	6.5 × 10^−4^
KB3.1	17.9	*	**	1.73 × 10^−5^

(*): Slight inhibition of cell proliferation, (**): no cytotoxic activity observed.

## Data Availability

The DNA sequences are deposited in GenBank (https://www.ncbi.nlm.nih.gov/genbank/ (accessed on 8 June 2023)) and all other relevant data are included in the Supplementary Information.

## Supplementary Materials

The following supporting information can be downloaded at: https://www.mdpi.com/article/10.3390/metabo13070848/s1, Table S1: ^1^H and ^13^C NMR data of panepophenanthrin (2); Table S2: ^1^H and ^13^C NMR data of dihydrohypnophilin (3); Figures S1 and S2: HPLC chromatogram and LR-/HRESIMS spectra of **1**; Figures S3–S6: 1D/2D NMR spectra of **1** in chloroform-*d*; Figures S7–S11: 1D/2D NMR spectra of **1** in methanol-*d_4_*:acetone-*d_6_* (3:1) at 500 (for ^1^H) and 125 (for ^13^C) MHz. Figure S12: Flow chart of the purification procedure.

## Sample Availability

Samples of compounds **2** and **3** are available from the authors. Sample of compound **1** is not available from the authors, due to consumption for structure elucidation and bioassays.
