# Supplementary material for: Panapophenanthrin, a Rare Oligocyclic Diterpene from Panus strigellus
Source: Metabolites. 2023 Jul 13;13(7):848. doi: 10.3390/metabo13070848 (PMC10385786; doi:10.3390/metabo13070848)
Supplement: Supplementary file 1 [file metabolites-13-00848-s001.zip › metabolites-2469715-SI.pdf]

Supplementary Materials for

Panapophenanthrin, a rare oligocyclic diterpene from *Panus strigellus*

Natalia A. Llanos-López [1,2], Sherif Saeed Ebada [1,3,*] Aida M. Vasco-Palacios [4], Laura M. Sánchez-Giraldo [5], Lina López [6], Luisa F. Rojas [6] Attila Mándi [7], Tibor Kurtán [7] and Yasmina Marin-Felix [1,2,*]

[1] Department of Microbial Drugs, Helmholtz Centre for Infection Research (HZI) and German Centre for Infection Research (DZIF), DZIF Partner Site Hannover-Braunschweig, Inhoffenstrasse 7, 38124 Braunschweig, Germany

[2] Institute of Microbiology, Technische Universität Braunschweig, Spielmannstraße 7, 38106 Braunschweig, Germany

[3] Department of Pharmacognosy, Faculty of Pharmacy, Ain Shams University, Cairo 11566, Egypt

[4] Grupo de Microbiología Ambiental and BioMicro, Escuela de Microbiología, Universidad de Antioquia, Calle 70 No. 52-21, 050010 Medellin, Colombia

[5] Grupo de Investigación de Biotecnología Industrial, Facultad de Ciencias, Universidad Nacional de Colombia Sede Medellín, Calle 59A No. 63-20, 050034 Medellin, Colombia

[6] Grupo de Biotransformación, Escuela de Microbiología, Universidad de Antioquia, Calle 70 No. 52-21, 050010 Medellin, Colombia

[7] Department of Organic Chemistry, University of Debrecen, P. O. Box 400, 4002 Debrecen, Hungary

* Correspondence: sherif.elsayed@helmholtz-hzi.de; sherif_elsayed@pharma.asu.edu.eg (S.S.E); yasmina.marinfelix@helmholtz-hzi.de (Y.M.-F.)

ABSTRACT

During the course of our search for biologically active secondary metabolites from fungal cultures, a new oligocyclic diterpenoidal derivative, panapophenanthrin (**1**), was isolated from *Panus strigellus*. In addition, two known metabolites, panepophenanthrin (**2**) and dihydrohypnophilin (**3**), were also obtained. The chemical structures of the isolated compounds were elucidated based on extensive 1D and 2D NMR spectral analyses together with high-resolution electrospray ionization mass spectrometry (HR-ESI-MS). The absolute configuration was determined through TDDFT-ECD calculations. All of the compounds were assessed for their antimicrobial and cytotoxic activities. Compounds **1** and **3** showed moderate to weak activities in the performed antimicrobial assays, while compound **1** exhibited potent cytotoxic activity against the mammalian cell lines mouse fibroblast (L929) and human endocervical adenocarcinoma (KB3.1).

Keywords: Basidiomycota; antimicrobial; cytotoxicity; secondary metabolites; white rot fungi.

Contents of Supporting Information

#	Contents	Page
**1**	Table S1. ^{1}H and ^{13}C NMR data of panepophenanthrin (**2**).	**S4**
**2**	Table S2. ^{1}H and ^{13}C NMR data of dihydrohypnophilin (**3**).	**S4**
**3**	Figure S1. HPLC chromatogram and LRESIMS spectrum of **1**.	**S5**
**4**	Figure S2. HPLC chromatogram and HRESIMS spectrum of **1**.	**S6**
**5**	Figure S3. ^{1}H NMR spectrum of **1** in chloroform-*d* at 500 MHz.	**S7**
**6**	Figure S4. ^{1}H-^{1}H COSY spectrum of **1** in chloroform-*d* at 500 MHz.	**S8**
**7**	Figure S5. HMBC spectrum of **1** in chloroform-*d* at 500 MHz.	**S9**
**8**	Figure S6. HSQC spectrum of **1** in chloroform-*d* at 500 MHz.	**S10**
**9**	Figure S7. ^{1}H NMR spectrum of **1** in methanol-d_4:acetone-d_6 (3:1) at 500 MHz.	**S11**
**10**	Figure S8. ^{13}C NMR spectrum of **1** in methanol-d_4:acetone-d_6 (3:1) at 125 MHz.	**S12**
**11**	Figure S9. HMBC spectrum of **1** in methanol-d_4:acetone-d_6 (3:1) at 500 MHz.	**S13**
**12**	Figure S10. HSQC spectrum of **1** in methanol-d_4:acetone-d_6 (3:1) at 500 MHz.	**S14**
**13**	Figure S11. ROESY spectrum of **1** in methanol-d_4:acetone-d_6 (3:1) at 500 MHz.	**S15**
**14**	Figure S12. Flow chart of the purification procedure.	**S16**

Table S1. ^{1}H and ^{13}C NMR data of panepophenanthrin (**2**).

pos.	δH (multi, *J*[Hz])[a]	δC, type[b]
1	4.21 (br s, 1H)	66.7, CH
2	3.38 (t, *J* = 3.5 Hz, 1H)	55.5, CH
3	3.23 (d, *J* = 4.1 Hz, 1H)	55.7, CH
3a		101.0, C
5		76.3, C
5a	3.09 (dd, *J* = 5.2, 1.7 Hz, 1H)	55.8, CH
6	6.64 (dd, 5.1, 2.9)	138.0, CH
6a		137.0, C
7		194.2, CO
8	3.44 (d, *J* = 4.1 Hz, 1H)	53.0, CH
9	3.79 (t, *J* = 3.5, 1H)	58.9, CH
10	4.45 (br s, 1H)	63.8, CH
10a	2.10 (d, 10.0, 1H)	50.1, CH
10b	1.79 (d, 10.0, 1H)	48.2, CH
10c		53.2, C
11	5.86 (d, *J* = 16.3 Hz, 1H)	127.2, CH
12	5.45 (d, *J* = 16.2 Hz, 1H)	140.9, CH
13		69.1, C
14	1.00 (s, 3H)	29.4, CH_3
15	1.05 (s, 3H)	30.2, CH_3
16	1.33 (s, 3H)	25.4, CH_3
17	1.24 (s, 3H)	31.8, CH_3
1-OH	5.13 (d, *J* = 5.2 Hz, 1H)	
3a-OH	5.96 (s, 1H)	
10-OH	4.12 (br s, 1H)	
13-OH	4.26 (br s, 1H)	

[a] Measured in DMSO-d_6 at 500 MHz.
[b] Assigned by HMBC and HSQC spectra.

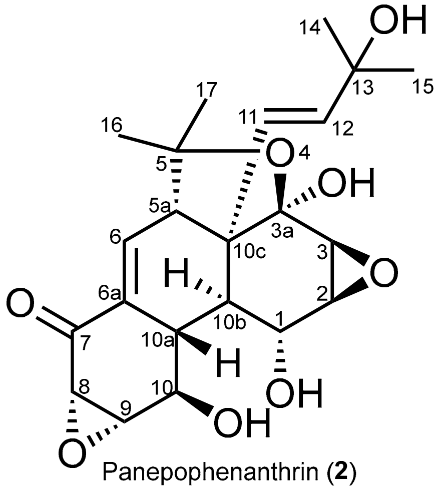

Panepophenanthrin (**2**)

Table S2. ^{1}H and ^{13}C NMR data of dihydrohypnophilin (**3**).

pos.	δH (multi, *J*[Hz])[a]	δC, type[b]
1	3.80 (d, 8.4, 1H)	81.0
2	2.02 (dd, 12.0, 8.4, 1H)	55.2
3		47.7
4		159.6
5	4.62 (d, 5.1, 1H)	74.2
6	3.48 (s, 1H)	63.8
7		75.1
8	1.83 d	30.7
9	2.61 (ddt, 19.9, 10.8, 8.5, 1H)	34.8
10	1.83 (dd, 12.9, 8.5, 1H)<br>1.14 (dd, 12.9, 10.8, 1H)	46.4
11		44.2
12	0.88 (s, 3H)	19.8
13	1.05 (s, 3H)	26.6
14	1.17 (s, 3H)	17.6
15	5.32 (d, 2.1, 1H)<br>5.15 (dd, 2.6, 0.8, 1H)	112.6

[a] Measured in chloroform-*d* at 500 MHz.
[b] Assigned by HMBC and HSQC spectra.

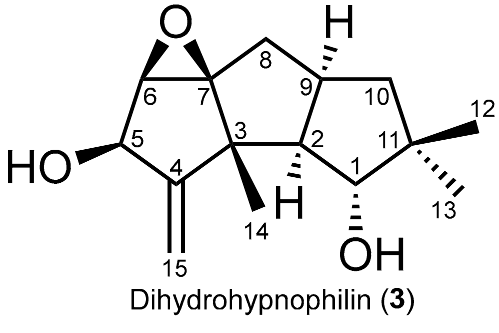

Dihydrohypnophilin (**3**)

Generic Display Report

Analysis Info

Analysis Name	S:\PEOPLE\nll21_Natalia Llanos\Panus\Panus (F5S3R2+F8S2R2)_F5_F4\Amazon\Panus (F5S3R2+F8S2R2)_F5_F4_BA8_01_39079.d	Acquisition Date	08.05.2022 00:09:32
Method		Operator	esu
Sample Name	Panus (F5S3R2+F8S2R2)_F5_F4	Instrument	amaZon speed
Comment			

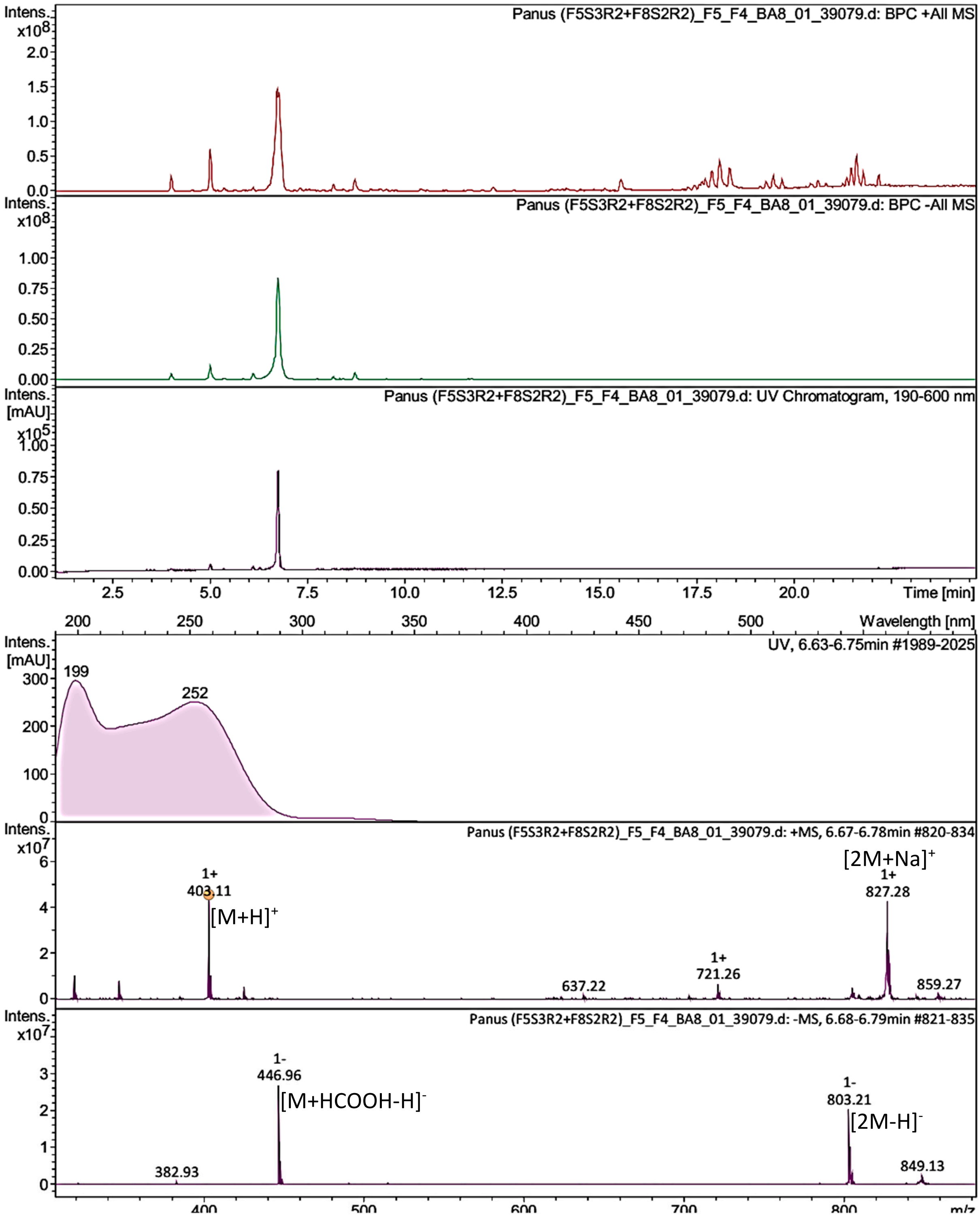

Bruker Compass DataAnalysis 4.4 printed: 07.05.2023 16:32:18 by: sel22 Page 1 of 1

Figure S1. HPLC chromatogram and LRESIMS spectrum of **1**.

Generic Display Report

Analysis Info Acquisition Date 19.05.2022 10:41:36

Analysis Name	C:\SEL22\Panus sp_Natalia\Panus (F5S3R2+F8S2R2)_F5_F4\Panus_F5S3R2+F8S2R2_F5_F4_P1-A-4_01_10193.d		
Method	pos_saure_1000RO_screening_ms_100-2500_final.m	Operator	ate06
Sample Name	Panus_F5S3R2+F8S2R2_F5_F4	Instrument	maXis
Comment	Screening01<br>Waters Acquity UPLC BEH C_18 1,7um 2.1x50mm		

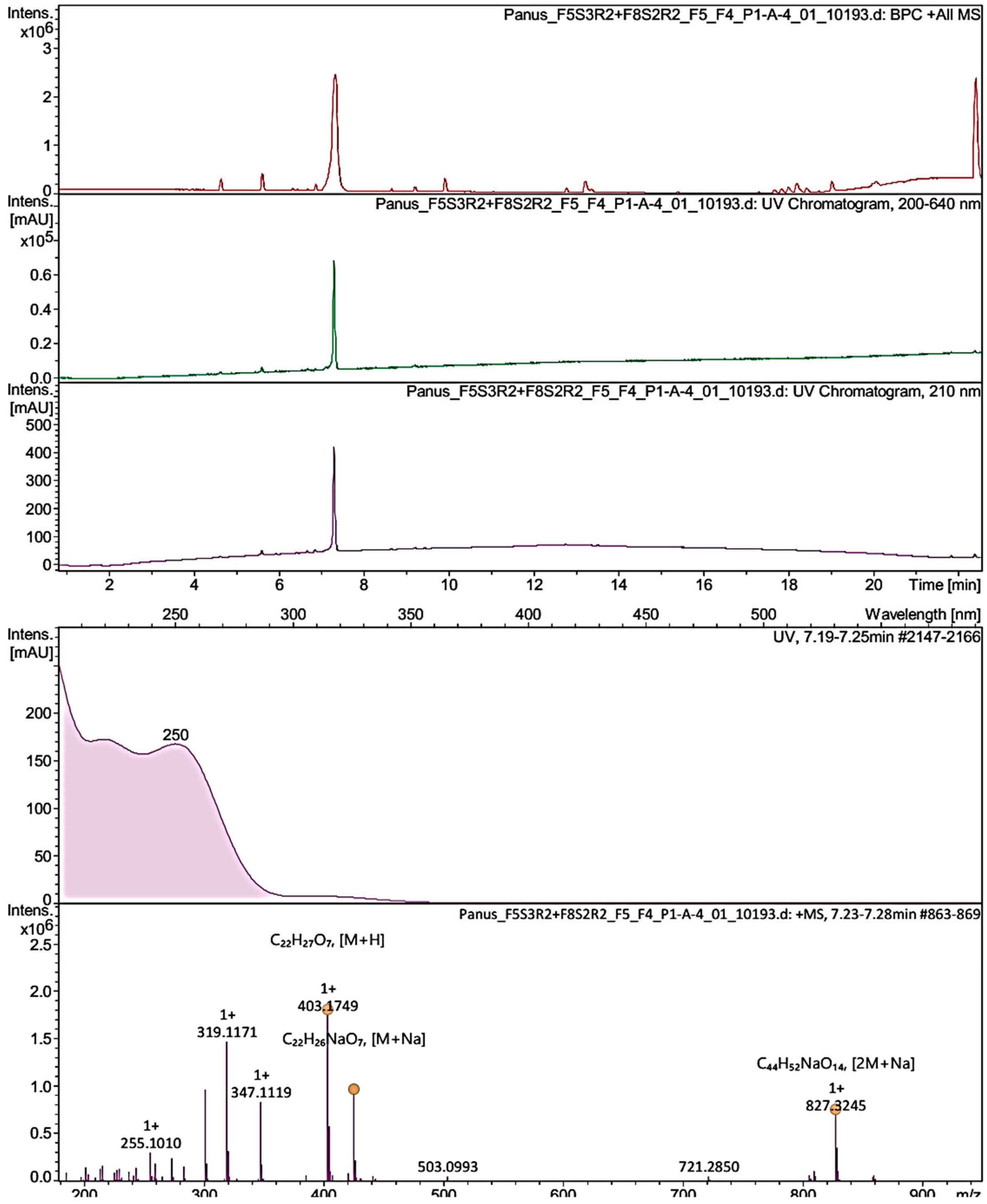

Figure S2. HPLC chromatogram and HRESIMS spectrum of **1**.

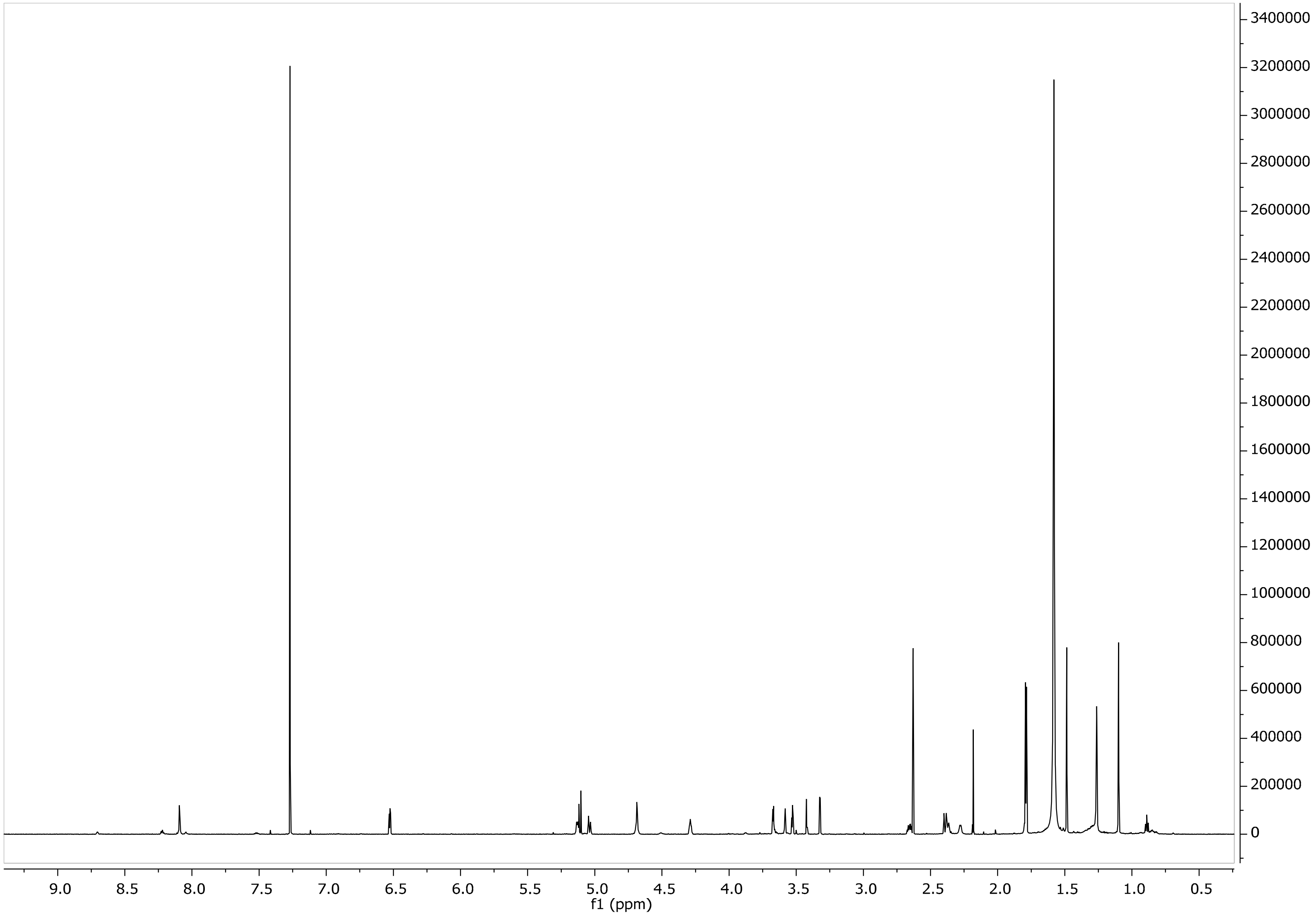

Figure S3. ^{1}H NMR spectrum of **1** in chloroform-*d* at 500 MHz.

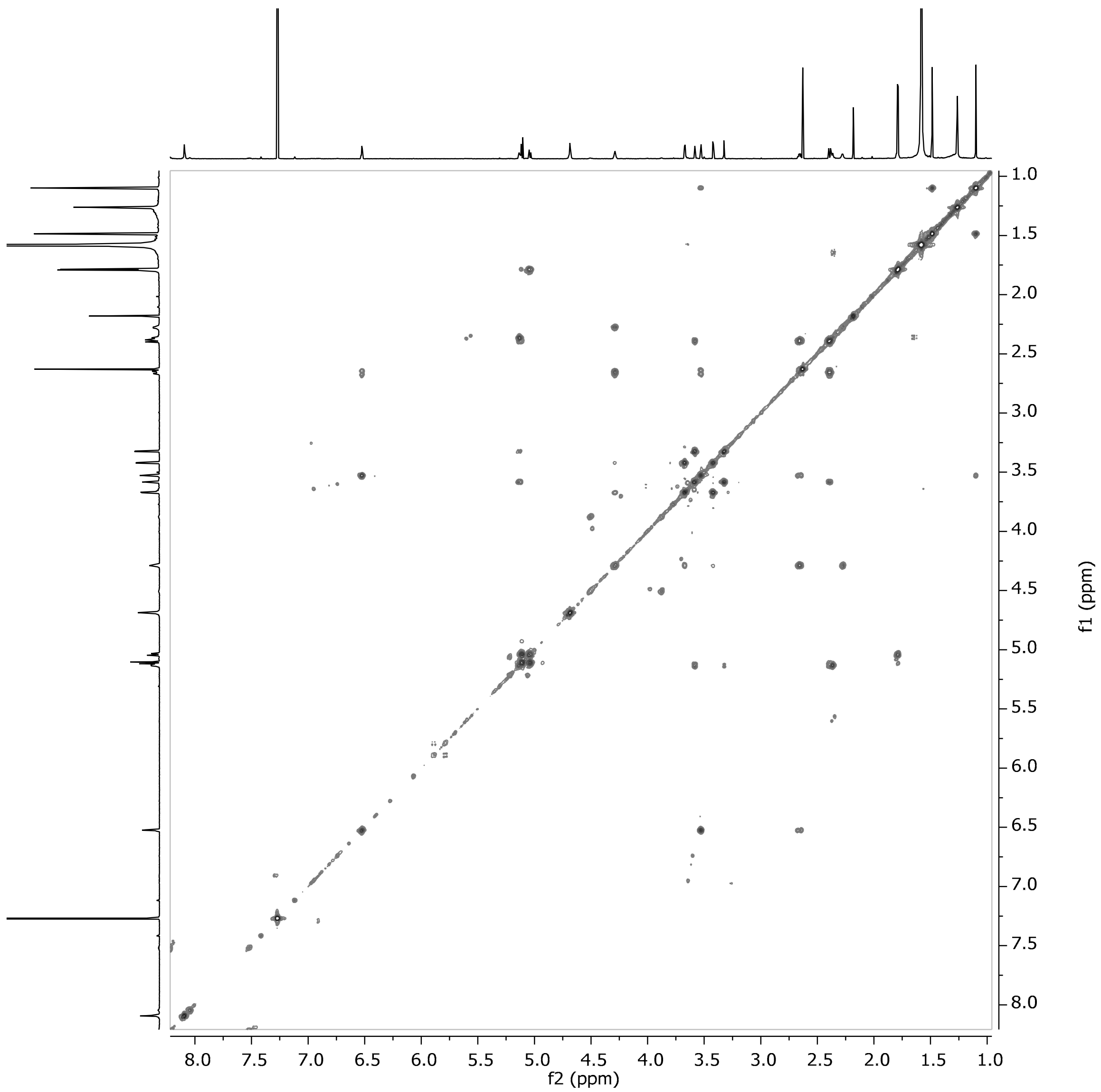

Figure S4. ^{1}H-^{1}H COSY spectrum of **1** in chloroform-*d* at 500 MHz.

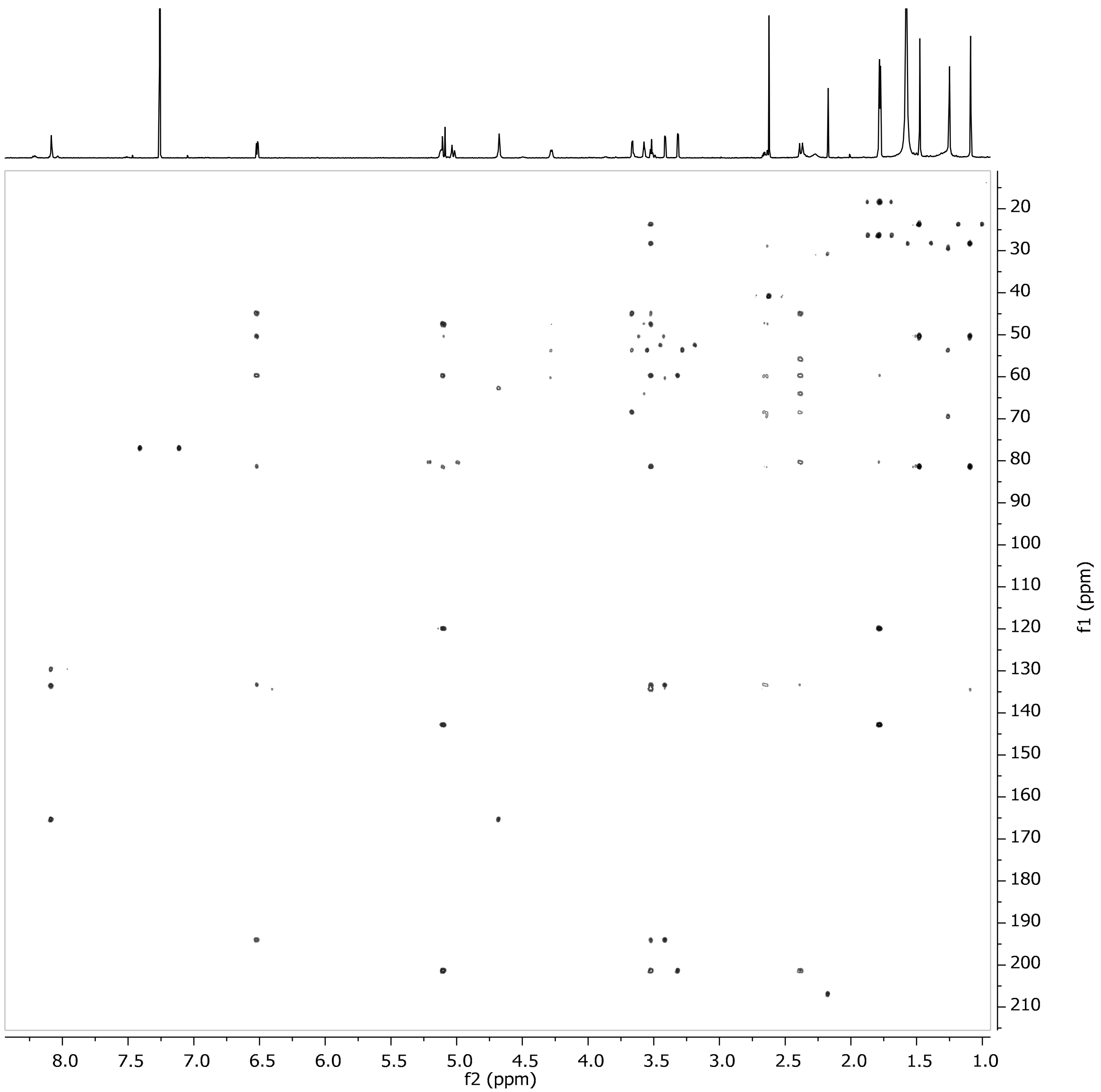

Figure S5. HMBC spectrum of **1** in chloroform-*d* at 500 MHz.

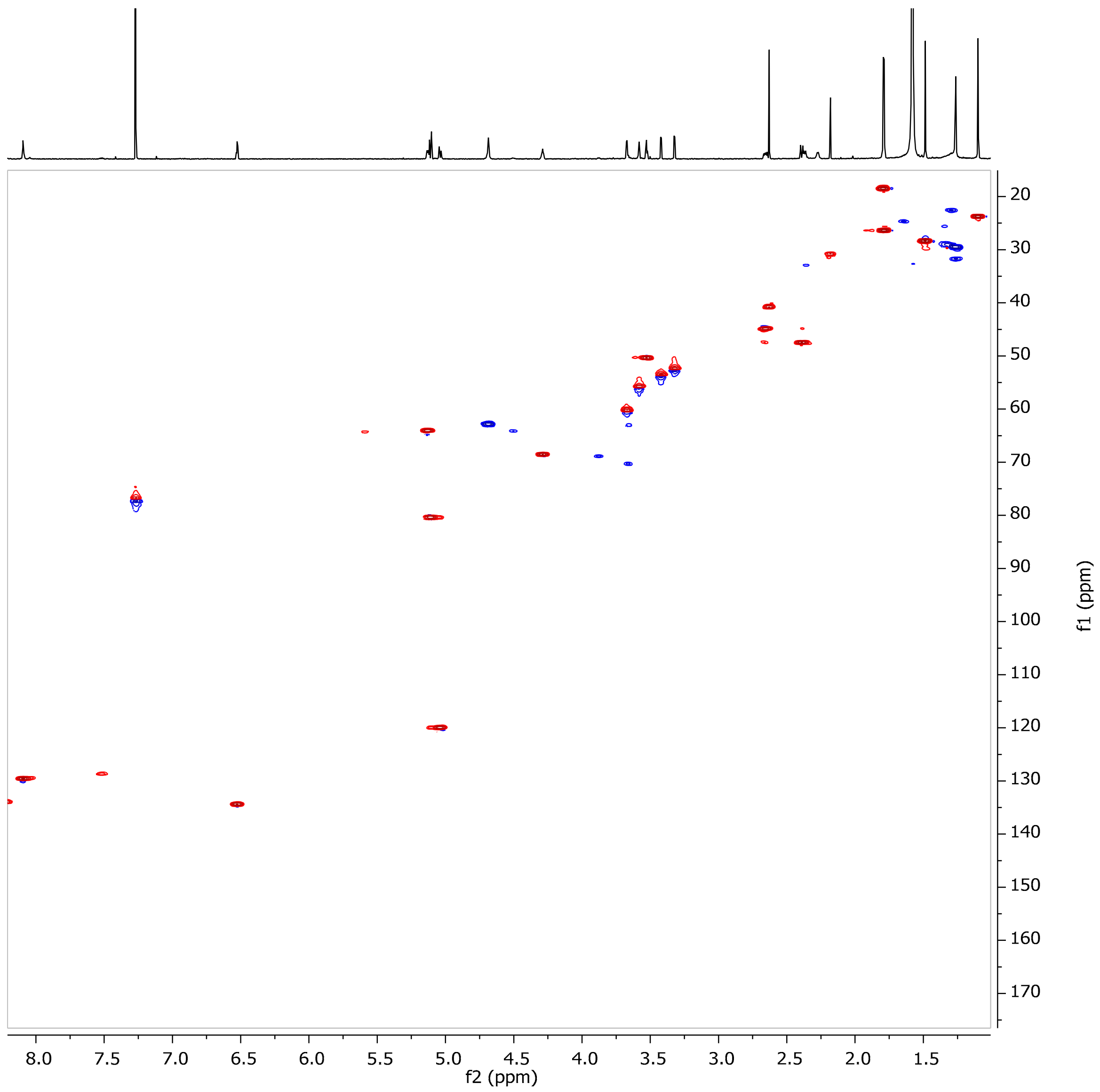

Figure S6. HSQC spectrum of **1** in chloroform-*d* at 500 MHz.

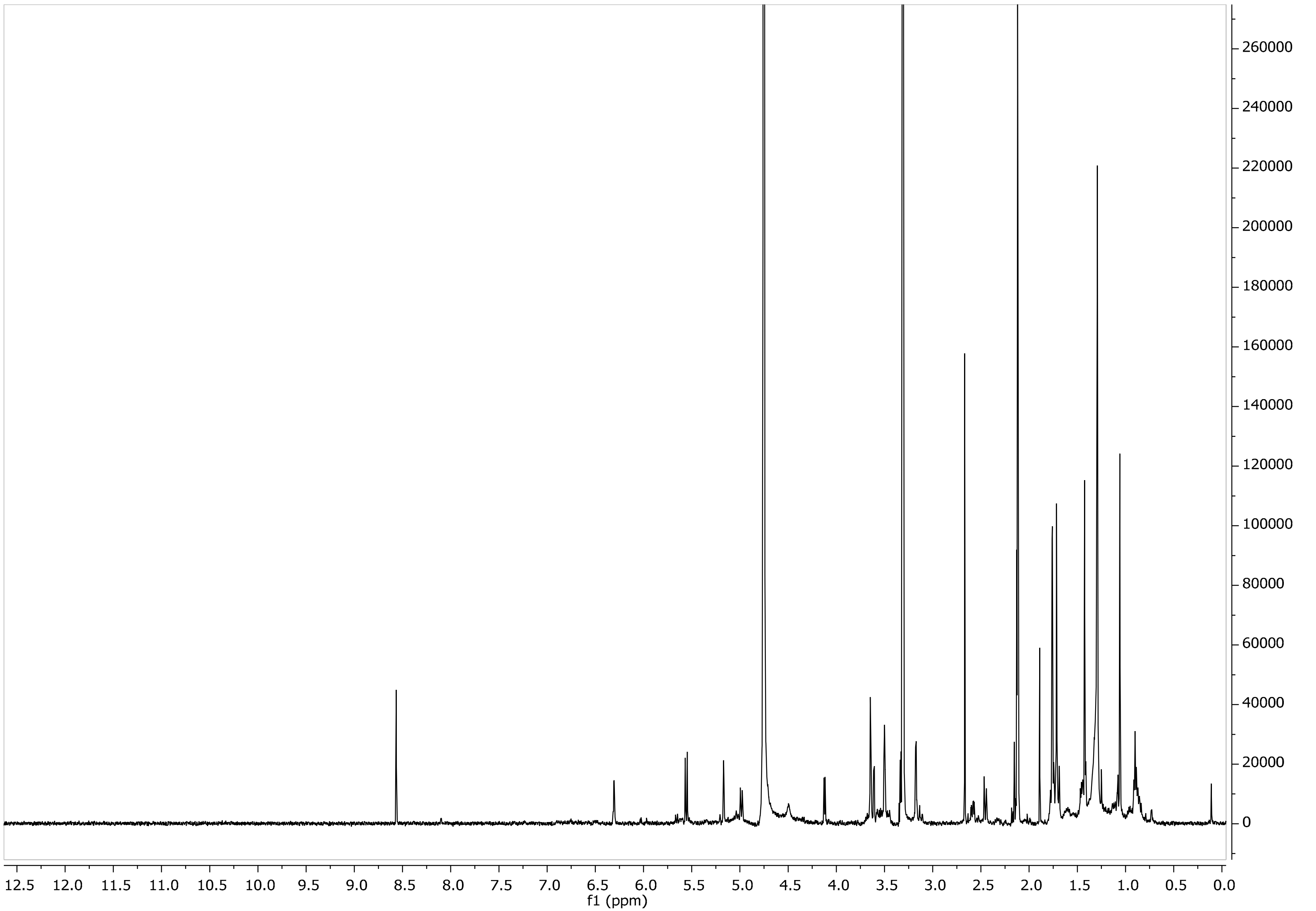

Figure S7. ^{1}H NMR spectrum of **1** in methanol-d_4:acetone-d_6 (3:1) at 500 MHz.

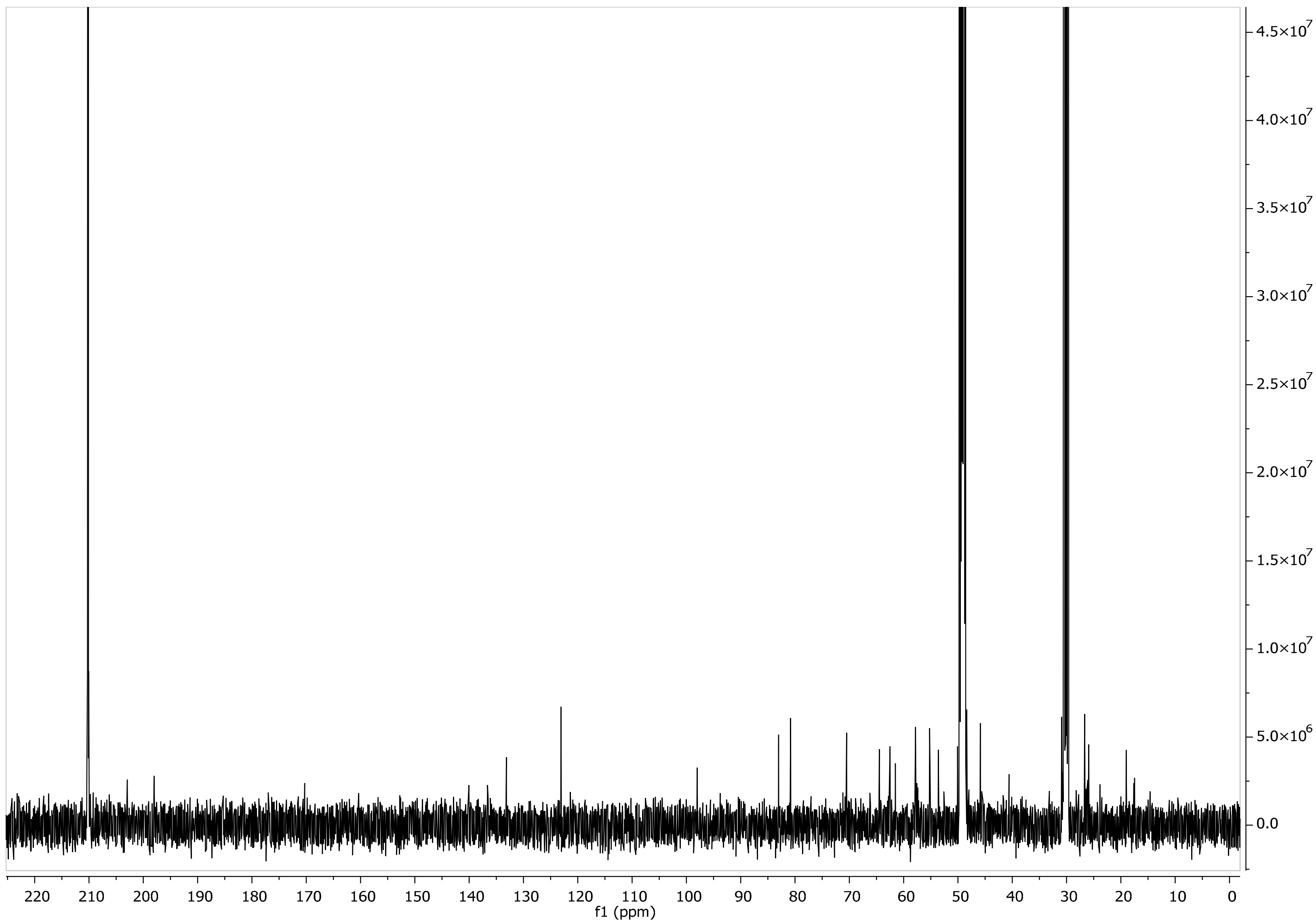

Figure S8. ^{13}C NMR spectrum of **1** in methanol-*d₄*:acetone-*d₆* (3:1) at 125 MHz.

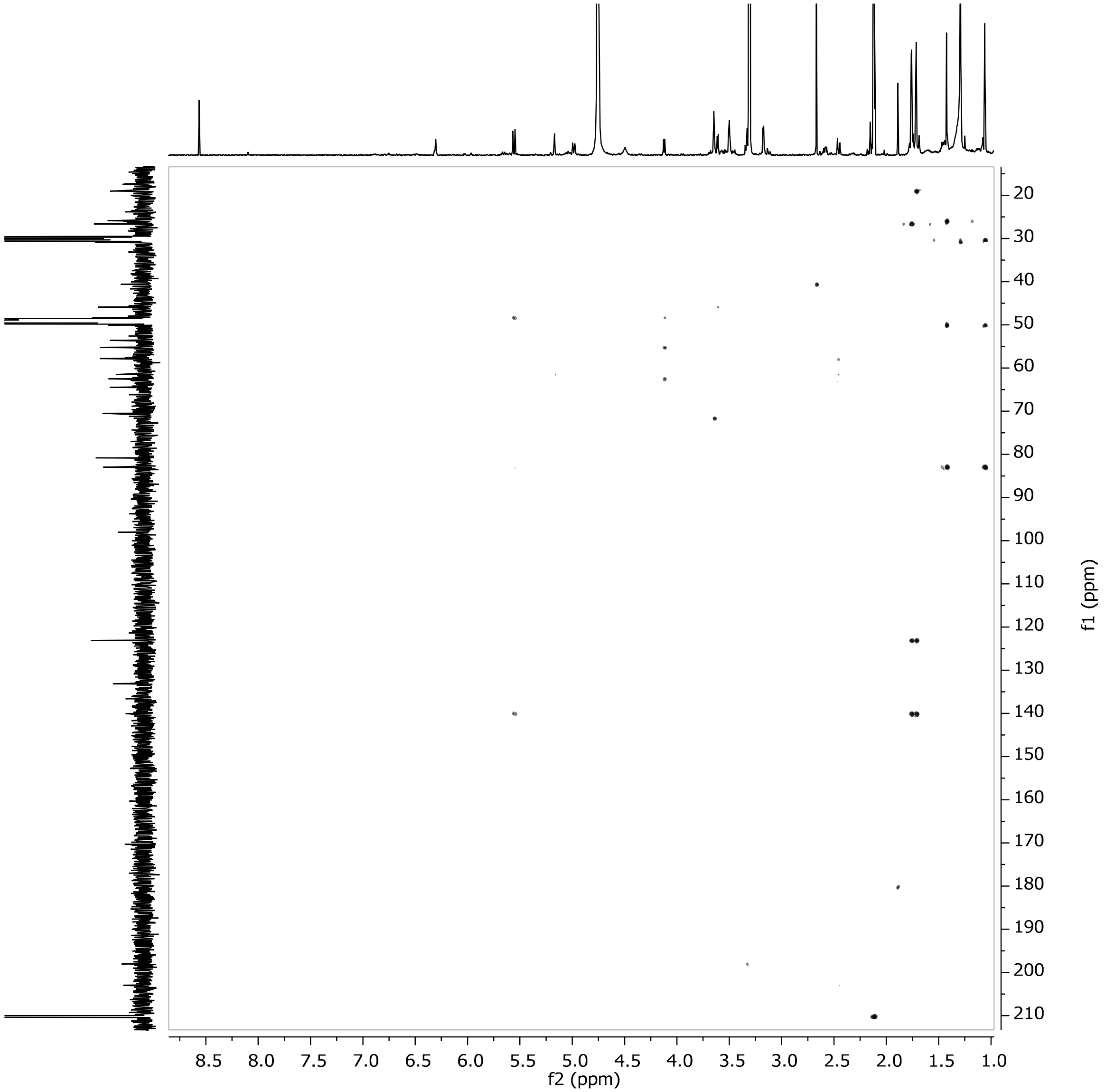

Figure S9. HMBC spectrum of **1** in methanol-*d4*:acetone-*d6* (3:1) at 500 MHz.

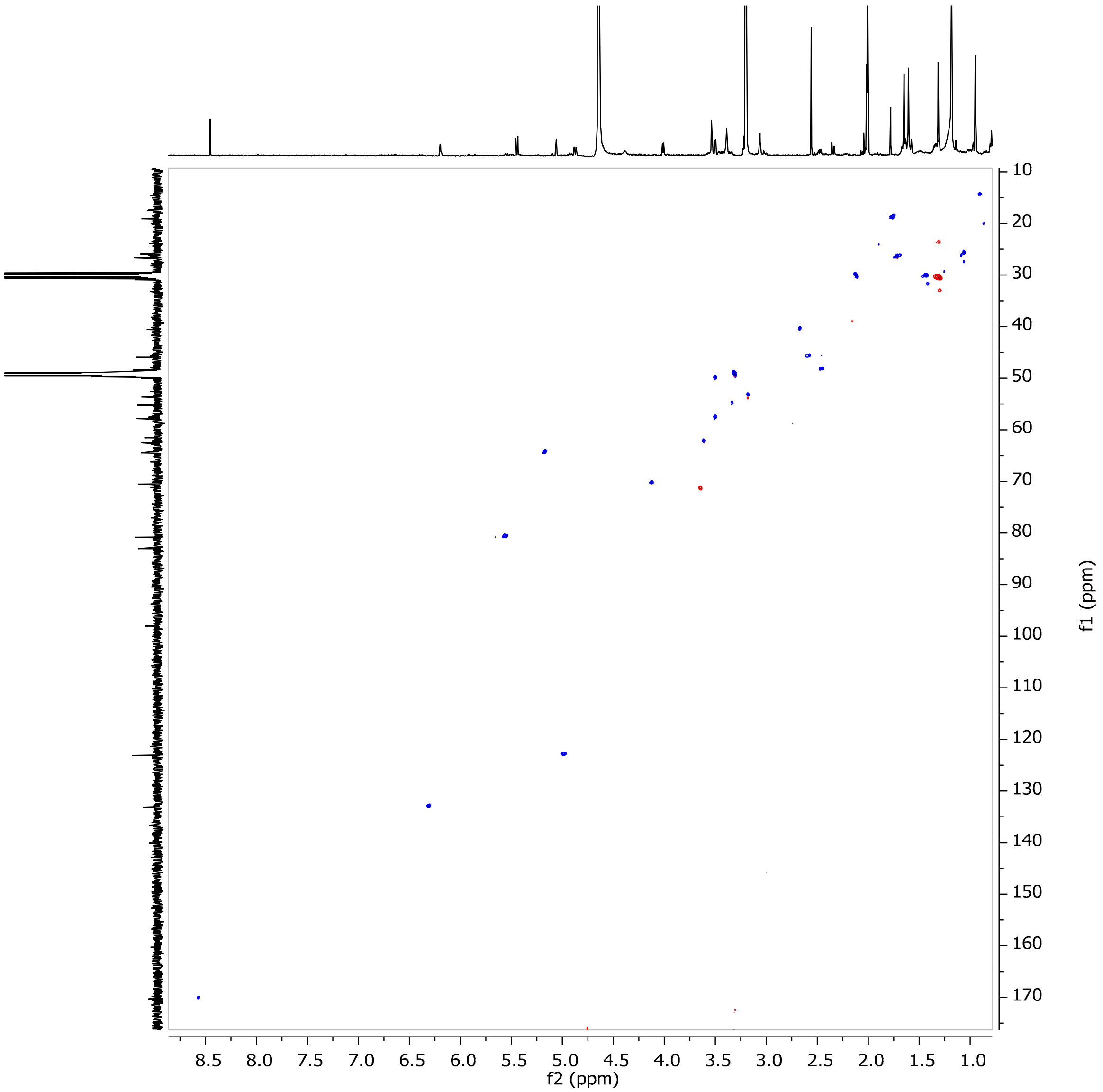

Figure S10. HSQC spectrum of **1** in methanol-d_4:acetone-d_6 (3:1) at 500 MHz.

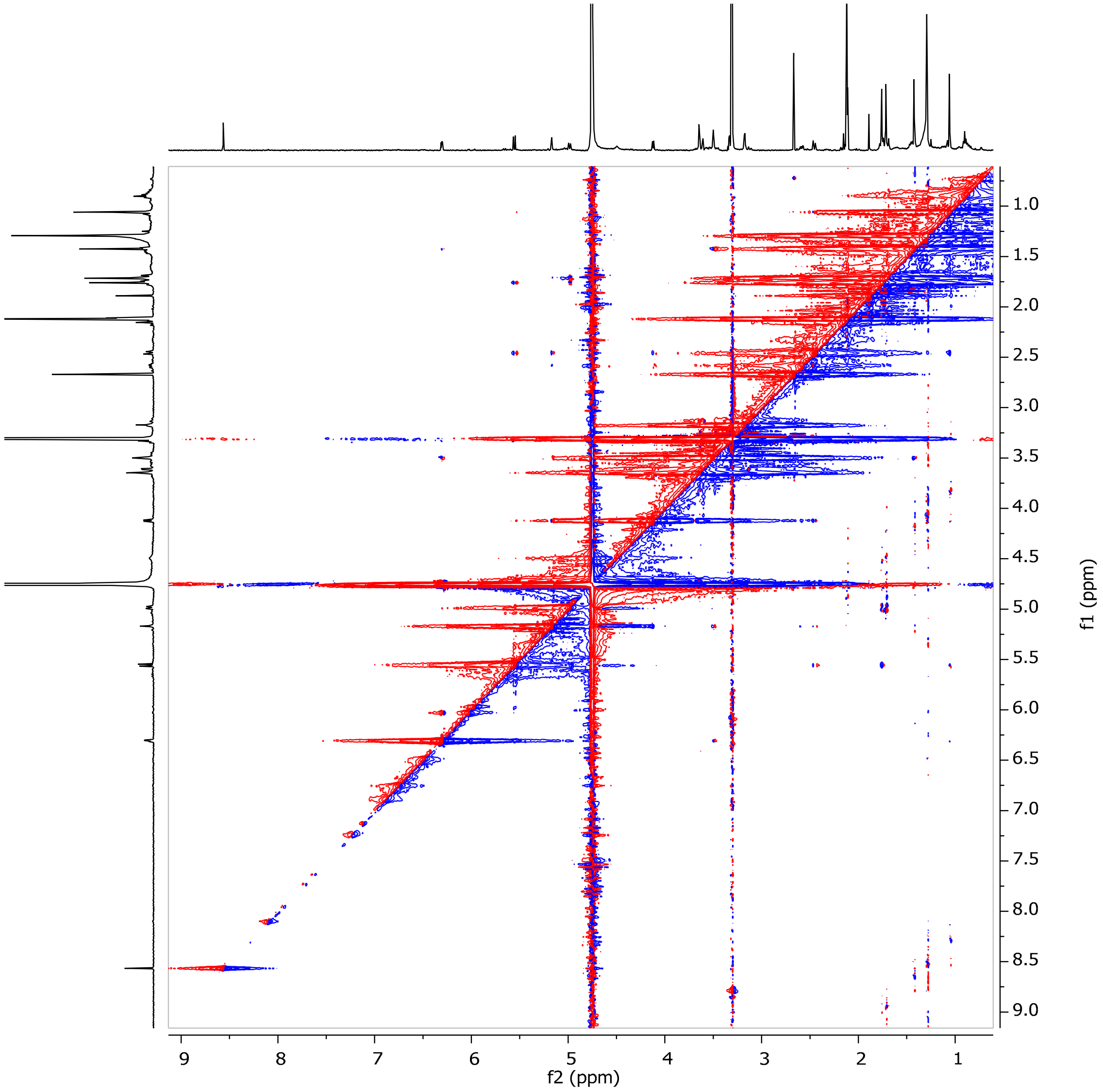

Figure S11. ROESY spectrum of **1** in methanol-d_4:acetone-d_6 (3:1) at 500 MHz.

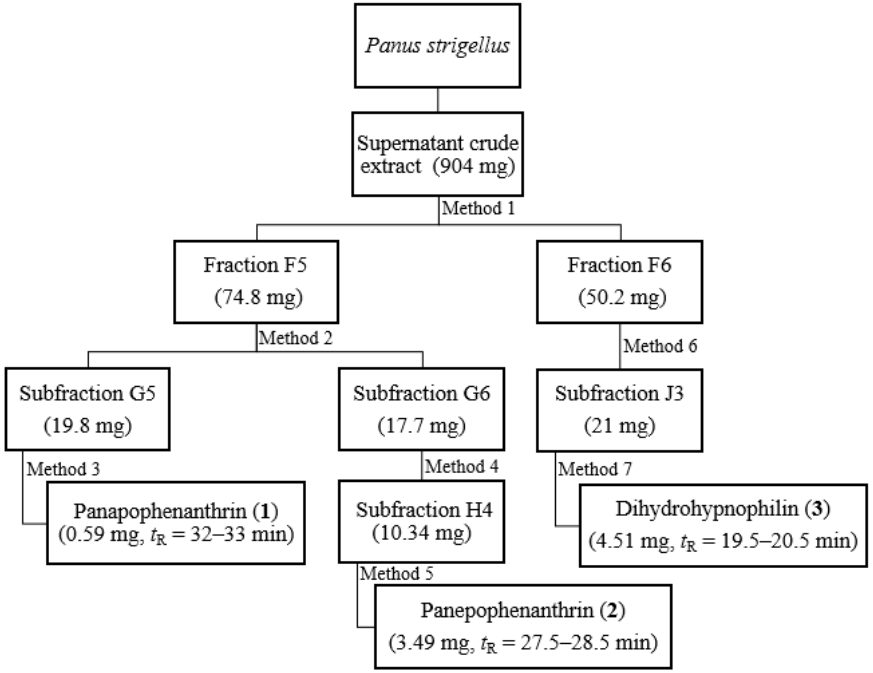

Method	Column	Flow rate (mL/min)	Gradient elution (B= MeCN with 0.1% Formic acid, up to 100% with A= H_2O with 0.1 % Formic acid)
1	Gemini® 10 µm C18 110 Å column (250 × 50 mm; Phenomenex, Torrance, CA, USA)	40	5% B for 8 min, 5% B to 20% B in 5 min, 20% B to 30% B in 30 min, 30% B to 42 %B in 30 min, 42 % B to 100% B in 5 min, and 100% B for 5 min
2	Gemini® 10 µm C18 110 Å column (250 × 21.2 mm; Phenomenex, Torrance, CA, USA)	20	10% B for 5 min, 10% B to 20% B in 10 min, 20% B to 25% B in 20 min, 25% to 40% B in 15 min, 40% B to 100% B in 5 min, and 100% B for 5 min
3	Synergi™ 10 µm Polar-RP 80Åcolumn (250×50 mm; Phenomenex, Torrance, CA, USA)	20	5% B for 3 min, 5% B to 15% B in 18 min, 15% B to 100% B in 25 min, and 100% B for 5 min
4	Synergi™ 10 µm Polar-RP 80Åcolumn (250×50 mm; Phenomenex, Torrance, CA, USA)	20	5% B for 3 min, 5% B to 50% B in 25 min, 50% B to 100% B in 8 min, and 100% B for 3 min
5	Luna® 5 µm C18 110 Å column (250 × 21.2 mm; Phenomenex, Torrance, CA, USA)	15	10% B for 3 min, 10% B to 25% B in 36 min, 25% B to 100% B in 3 min, and 100% B for 3 min
6	Gemini® 10 µm C18 110 Å column (250 × 21.2 mm; Phenomenex, Torrance, CA, USA)	20	25% B for 5 min, 25% B to 45% B in 20 min, 45% B to 100% B in 10 min, and 100% B for 5 min
7	XBridge 5 µm C18 column (250 × 19 mm; Waters, Milford, MA, USA)	20	5% B for 3 minutes, 5% B to 20% B in 2 min, 20% B to 30% B in 30 min, 30% B to 100% B in 5 min, and 100% B for 3 min

Figure S12. Flow chart of the purification procedure
